# High-affinity IgE receptor-β chain expression in human mast cells

**DOI:** 10.1016/j.jim.2008.05.006

**Published:** 2008-07-31

**Authors:** Akira Matsuda, Yoshimichi Okayama, Nobuyuki Ebihara, Norihiko Yokoi, Peisong Gao, Junji Hamuro, Julian M. Hopkin, Shigeru Kinoshita

**Affiliations:** a Department of Ophthalmology, Kyoto Prefectural University of Medicine, Kyoto, Japan; b Division of Molecular Cell Immunology and Allergology, Advanced Medical Research Center, Nihon University Graduate School of Medicine, Tokyo, Japan; c Department of Ophthalmology, Juntendo University School of Medicine, Tokyo, Japan; d Johns Hopkins Asthma and Allergy Center, Johns Hopkins University School of Medicine, Baltimore, MD, USA; e Experimental Medicine Unit, University of Wales Swansea, Swansea, UK

**Keywords:** FcεRI, high-affinity IgE receptor, b-mast cells, bone-marrow-derived mast cells, Atopic diseases, IgE receptor, Immunoblotting, Immunocytochemistry, Mast cells

## Abstract

The high-affinity IgE receptor (FcεRI)-β gene is one of the atopy-associated genes, but its biological significance is largely unknown. In this study, we generated the anti-FcεRI-β chain antibody to clarify β-chain protein expression in human mast cells. The FcεRI-β antibody showed specific binding to a 27 kDa protein with Western blotting and membrane bound immunostaining using cultured mast cells. Monomeric IgE sensitization increased β-chain expression as well as mature α-chain expression in mast cells. Upregulation of β-chain expression with monomeric IgE treatment suggests possible roles of FcεRI-β protein as an atopy-related molecule.

## 1 Introduction

The high-affinity IgE receptor (FcεRI)-β has been recognized as an atopy-related gene (Cookson et al., 1989 and Shirakawa et al., 1994). Some lines of study using a mouse reconstitution system suggested that the FcεRI-β protein may act as an amplifier for mast mast-cell activation (Lin et al., 1996). Mouse models are often useful tools for biomedical research, but in the case of FcεRI-β, there are significant discrepancies between the mouse and human system. For example, FcεRI-β protein expression is essential for cell-surface expression of FcεRI in mice (Turner and Kinet, 1999), however, human dendritic cells showed a cell-surface FcεRI receptor without β-chain mRNA expression (Bieber et al., 1996). Therefore, the existence of FcεRI without β-chain expression (FcεRI-αγ_2_ subtype) is probable in humans, but not in mice (Hayashi et al., 1999). However, we could not distinguish FcεRI-αβγ from FcεRI-αγ_2_ in situ due to the lack of reliable anti-human FcεRI-β antibodies for histochemical use. We chose to raise polyclonal rabbit antibodies to a specific peptide of human FcεRI-β in order to help the identification of FcεRI-β protein. One such antibody preparation successfully bound to the expected 27 kDa band on immunoblotting using human mast-cell/basophil lysate.

Another problem related to the functional study of human FcεRI-β protein is a lack of good human cell lines which express FcεRI. Human peripheral-blood-derived basophils and mast cells had been so far recognized as the source for FcεRI, however, the amounts of the cells were practically not sufficient for protein analysis. Recently, Kirshenbaum et al. (2003) had established a cell line (LAD2) from a human mastocytoma patient, which retains the character of native human mast cells and expresses functional FcεRI. Using the LAD2 cell line, we further proceeded to verify the specificity of the antibody with immunoblotting and immunoprecipitation studies. The new antibody reacted with FcεRI-β protein and was useful for immunoblotting and immunocytochemical staining.

## 2 Materials and methods

### 2.1 Antibodies

A rabbit anti-serum against unique C-terminal sequences of FcεRI-β (CYSELEDPGEMSPPIDL) was generated by Affinity Research Products Ltd. (Exeter, UK). The anti-serum was purified on a protein-A column (Amersham Plc., Little Chalfont, UK). Other antibodies used in this study included Alexa 488-goat anti-rabbit-F(ab′)_2_ and Alexa 594-goat anti-mouse IgG-F(ab′)_2_ (Invitrogen, Carlsbad, CA, USA), chimeric anti-NIP IgE antibody (Serotec, Oxford, UK), rabbit anti-FcεRI-α and rabbit anti-FcεRI-γ polyclonal antibodies (Upstate Biotechnology, Lake Placid, USA), and mouse anti-FcεRI-α monoclonal antibody (clone:CRA1; Kyokuto Pharmaceuticals, Tokyo, Japan).

### 2.2 Reagents

Reagents used in this study included Kaleidoscope Prestained Protein Standards (Bio-Rad Japan, Tokyo, Japan), Tris–glycine gels (Invitrogen), sodium dodecyl sulfate (SDS), dl-dithiothreitol (DTT), and phosphatidylserine, alcian blue dye (Sigma Japan, Tokyo, Japan), 3-Cyclohexylamino-1-propanesulfonic acid (CAPS; Dojindo, Kumamoto, Japan), complete mini protease cocktail tablet (Roche Ltd., Penzberg, Germany), and HCL Plus Western blotting detection reagents (Amersham). All other reagents used in this study were of analytical grade.

### 2.3 Cell culture

Human basophils and eosinophils were purified from venous blood with a basophil isolation kit or with anti-CD16 beads, respectively, with Midi MACS (Miltenyi Biotec, Gladbach, Germany). Bone-marrow-derived CD34 + cells were purchased from Cambrex North Brunswick, Inc. (North Brunswick, NJ) and bone-marrow-derived mast cells (b-mast) were generated as previously described (Saito et al., 2006). Human blood samples were collected from volunteers with written informed consents, and all procedures were approved by the ethical committees of Kyoto Prefectural University of Medicine and in accordance with the Declaration of Helsinki. Purity of the basophils and eosinophils was checked with alcian blue staining and Hansel staining (Eosino-Stain; Tori-Pharmaceuticals, Tokyo, Japan), respectively, and each showed a > 98% purity. Human mast mast-cell line LAD2 was kindly provided by Dr. Arnold Kirshenbaum (NIAID, NIH) and maintained as previously described (Kirshenbaum et al., 2003).

### 2.4 SDS-polyacrylamide gel electrophoresis (PAGE) and Western blotting

LAD2 cells, eosinophils, and basophils were collected, washed twice with phosphate-buffered saline (PBS), and the number of cells was counted. Cells in the amount of 2 × 10^4^ were then solubilized in SDS-sample buffer (62.5 mM Tris–HCl, pH6.8, 2%SDS, 20% Glycerol, and 0.04% bromophenol blue). Next, 50 mM DTT was added to the samples and incubated 15 min at 65 °C. Of each sample, 15 ml was loaded to 12% Tris–glycine gel with Kaleidoscope prestained protein standards. SDS-PAGE and Western blotting were then performed. The electrophoresed protein was transferred to polyvinylidene fluoride (PVDF) membrane (Pall Japan, Tokyo, Japan) using CAPS transfer buffer (10 mM CAPS, pH8.6, and 10% methanol). The membrane was then incubated with primary antibodies (1:1000 dilution in 1% non-fat skim milk) overnight at 4 °C with constant agitation. After washing with PBS containing 0.05% Tween 20 (PBST), the membrane was incubated with a 1:10,000 dilution of HRP conjugated anti-rabbit IgG. (Amersham) for 1 h and then visualized with ECL Plus Western blotting reagents.

### 2.5 Immunoprecipitation and subsequent immunoblotting

LAD2 cells in the amount of 2 × 10^7^ were collected and washed twice with PBS, and then solubilized with 2 ml of lysis buffer (2 mM l-α-phosphatidylcholine, 10 mM CHAPS, 150 mM NaCl, and 50 mM Tris Tris–HCl pH 8.0 with a complete mini protease inhibitor tablet) and centrifuged at 15,000 rpm for 30 min at 4 °C. The supernatant of the LAD2-cell lysate was pre-cleared with protein-G sepharose for 30 min. Then the supernatant was separated in half, and 1 μg of mouse anti-FcεRI-α monoclonal antibody or the same amount of isotype-matched control (mouse IgG2a) was added to each supernatant. After a 3-hour incubation at room temperature (RT), protein-G sepharose was added and further incubated for 1 h. The protein-G beads were washed 5 times with lysis buffer, then immunoprecipitated protein was solubilized with 50 μl of SDS-sample buffer. The immunoprecipitated protein samples were immunoblotted with anti-FcεRI-β and -γ antibodies as described above.

### 2.6 Flowcytometry and confocal microscopy analysis

LAD2, b-mast cells, and eosinophils were washed with PBS twice and fixed with 4% paraformaldehyde (PFA) in PBS for 15 min on ice. After PBS washing, the cells were permeabilized with 0.1% saponin in blocking buffer (10% normal goat serum, 1% bovine serum albumin in PBS) for 30 min on ice. Non-specific Fc receptor binding was blocked using an Fc receptor blocking reagent (Miltenyi Biotec). The cells were then washed again with PBS and incubated with anti-FcεRI-β IgG antibody (at the concentration of 20 μg/ml in blocking buffer) for 15 min at RT. As a negative control, the same amount of rabbit IgG (Santa Cruz Biotechnology, Inc., Santa Cruz, CA, USA) was used. After PBS washes, the samples were incubated with 1 μg/ml of Alexa 488 conjugated anti-rabbit antibody for 30 min. The stained LAD2 cells were also analyzed with a FACS caliber (Becton Deckinson Japan, Tokyo, Japan). For double-staining with FcεRI-α, the fixed LAD2 and b-mast cells were treated with Fc receptor blocking reagents, then incubated with mouse anti-FcεRI-α monoclonal antibody and post-fixed with 4% PFA-PBS. The cells were then permeabilized with saponin and incubated with the anti-FcεRI-β antibody. After PBS washes, the cells were reacted with Alexa 488 conjugated anti-rabbit IgG and Alexa 594 conjugated anti-mouse IgG simultaneously. The stained cells were then visualized with a confocal microscope (Fluoview 300; Olympus, Tokyo, Japan).

### 2.7 IgE sensitization of LAD2 cells and quantification of the FcεRI-β protein

LAD2 cells were sensitized with 0.5 μg/ml or 1.0 μg/ml of NIP-IgE antibody for 24 h. After sensitization, the cells are collected, washed twice with PBS, and the number of cells was counted. Cells in the amount of 4 × 10^4^ were then solubilized in SDS-sample buffer. SDS-PAGE and Western blotting was were carried out either with rabbit anti-FcεRI-α or anti-FcεRI-β polyclonal antibodies as described earlier. We quantified the intensity of FcεRI-β positive bands by densitometry (NIH image).

### 2.8 Reverse-transcription (RT) and real-time PCR analysis

Total RNA was isolated with the NucleoSpin RNA II extraction kit (Macherey-Nagel, Duren, Germany) from LAD2 cells, and then cDNA was prepared as described previously (Shimizu et al., 2005). We used real-time PCR probes and primers (Assay-on-Demand gene expression products; Applied Biosystems, Foster City, CA, USA) specific for human FcεRI-β(Hs00175091_m1) and FcεRI-α(Hs00758599_m1), and GAPDH. Real-time PCR analysis was carried out on a PRISM 7300 sequence detection system (Applied Biosystems). The relative expression of FcεRI mRNA in LAD2 cells was quantified by the standard curve method using GAPDH expression in the same cDNA as a control.

## 3 Results

### 3.1 Western blotting of cell lysates with the anti-FcεRI-β antibody

**Fig. 1:**
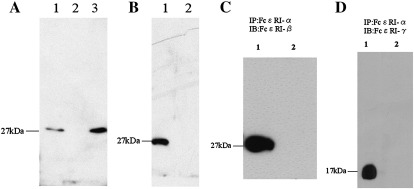
Immunoblotting and immunoprecipitation with the anti-FcεRI-β antibody. Immunoblotting was carried out with the anti-FcεRI-β antibody (A and B). The 27 kDa single band was observed with samples from basophils and LAD2 lysates, but not with eosinophils (A). Lane 1: basophil; Lane 2: eosinophil; Lane 3: LAD2. Electrophoresed LAD2-cell lysates were transferred to the same PVDF membrane and split into two pieces. One half (Lane 1) was incubated with the anti-FcεRI-β antibody, and the remaining half (Lane 2) was incubated with the FcεRI-β antibody preincubated with the immunized peptide (B). Immunoprecipitation and subsequent Western blotting was performed (C and D). LAD2-cell lysate was immunoprecipitated with the anti-FcεRI-α chain (CRA-1) antibody or isotype-matched IgG2a antibody. The samples were electrophoresed in a non-reducing condition and transferred onto a PVDF membrane. Western blotting was performed with either the anti-FcεRI-β (C) or anti-FcεRI-γ chain (D) antibodies. Lane 1: a sample immunoprecipitated with the anti-FcεRI-α monoclonal antibody; Lane 2: a sample immunoprecipitated with the control antibody.

The 27 kDa single band was detected with basophil lysate and LAD2 lysate, but not with eosinophil lysate (Fig. 1A). We examined basophils and eosinophils from three healthy volunteers. Although there was some variation of the FcεRI-β protein protein-expression levels in basophils from subject to subject, the results were essentially the same as this result (data not shown). The detection was completely ablated with the pre-incubation of the FcεRI-β antibody with a corresponding peptide (Fig. 1B). Preimmune serum of the same rabbit did not show any non-specific binding (data not shown).

### 3.2 Immunoprecipitation and subsequent immunoblotting

The FcεRI-α immunoprecipitates were immunoblotted with the anti-FcεRI-β antibody (Fig. 1C) and anti-FcεRI-γ polyclonal antibody (Fig. 1D), and the corresponding single band was observed by each blotting. No bands were observed with the control sample immunoprecipitated with the isotype-matched antibody.

### 3.3 Flowcytometry analysis and immunocytochemistry

**Fig. 2:**
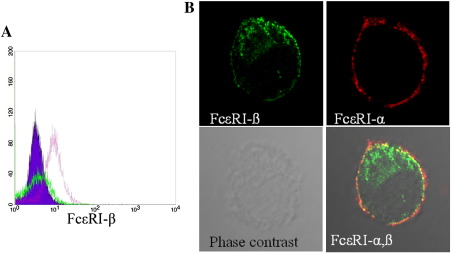
Flowcytometry and immunocytostaining with the anti-FcεRI-β antibody. (A) LAD2 cells were fixed, permeabilized with saponin, and immunostained with the anti-FcεRI-β antibody and Alexa 488 conjugated anti-rabbit IgG antibody. Normal rabbit IgG was used as a primary antibody for control staining (purple), anti-FcεRI-β staining with saponin treatment (red), and anti-FcεRI-β staining without saponin treatment (green). Immunocytostaining with anti-FcεRI-β and -α antibodies (B). Staining with FcεRI-β (green: upper left) staining and FcεRI-α (red: upper right), phase-contrast images (lower left) were merged with anti-FcεRI-α/-β double-staining (lower right).

**Fig. 3:**
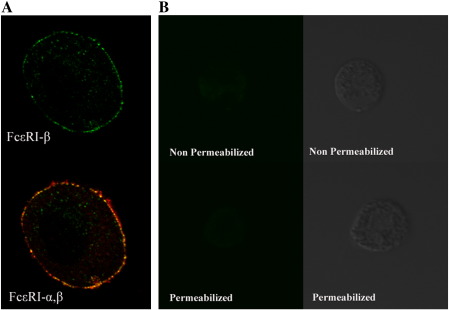
Immunocytostaining using b-mast cells and eosinophils with the anti-FcεRI-β antibody. B-mast cells were immunostained with anti-FcεRI-β antibody (upper) and double immunostained with anti-FcεRI-α (red) and -β (green) antibody (lower) (A). No positive anti-FcεRI-β immunostaining was observed using eosinophils without permeabilization (upper left) or with permeabilization (lower left); phase-contrast images are shown at the right side (B).

LAD2 cells with saponin treatment incubated with the FcεRI-β antibody showed positive staining by flowcytometry analysis compared to the negative control antibody staining. The fixed cells without saponin treatment showed negative staining with the FcεRI-β antibody (Fig. 2A). Immunostained LAD2 cells were also visualized by confocal microscopy, which showed a membranous positive staining with the FcεRI-α monoclonal antibody without cell permeabilization (Fig. 2B, upper left). FcεRI-β staining needed subsequent saponin treatment, and it showed membranous as well as some cytoplasmic staining (Fig. 2B, upper right). Double-immunostaining using the FcεRI-β and the monoclonal FcεRI-α antibody showed membranous colocalization (Fig. 2B, lower right). Double-immunostaining with b-mast cells showed a similar staining pattern to that of LAD2 cells (Fig. 3A). To rule out non-specific immunostaining with the anti-FcεRI-β antibody after cell permeabilization, we checked the immunostaining of eosinophils with and without saponin treatments. No definite immunostaining was observed even with permeabilized eosinophils (Fig. 3B).

### 3.4 The effect of monomeric IgE for FcεRI subunit expression

**Fig. 4:**
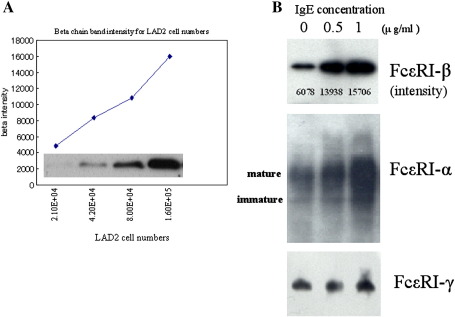
Quantification of FcεRI subunit proteins by immunoblotting. Different numbers of LAD2 cells (2.1 × 10^4^ cells to 1.6 × 10^5^ cells, two-fold gradients) were electrophoresed and immunoblotted with the anti-FcεRI-β antibody, and the intensity of the FcεRI-β positive band was quantified by densitometry (NIH image; Fig. 2A). LAD2 cells were preincubated with monomeric IgE for 24 h, 4 × 10^4^ cells were electrophoresed, then FcεRI-β, -α, and -γ protein expression was examined by immunoblotting (Fig. 2B). Lane 1: naïve LAD2 cells; Lane 2: preincubated with 0.5 μg/ml IgE; Lane 3: preincubated with 1 μg/ml IgE.

The protein gradient was made according to the number of LAD2 cells, and we found the linearity of FcεRI-β protein immunoblotting at the range of 2.1 × 10^4^ cells to 1.4 × 10^5^ cells (Fig. 4A). Then, the effect of monomeric IgE incubation for FcεRI-β protein expression was examined. FcεRI-β protein expression increased at 24 h after IgE incubation (Fig. 4B). Coordinated FcεRI-α upregulation was also found, but no difference for FcεRI-γ expression.

Real-time PCR analysis was also carried out to quantify FcεRI-β and FcεRI-α mRNA expression at 24 h after monomeric IgE (1.0 μg/ml) stimulation. The relative FcεRI-β mRNA expression ratio (monomeric IgE stimulation/mock stimulation) was 1.017 ± 0.109 (mean ± SD) and the relative FcεRI-α mRNA expression ratio was 0.726 ± 0.027. The result is a representative result of two independent experiments run in triplicate.

## 4 Discussion

We raised the anti-FcεRI-β polyclonal antibody against its C-terminal peptides and confirmed the specificity. At first we made two anti-peptide polyclonal antibodies against both N-terminal and C-terminal peptide sequences of the FcεRI-β protein, respectively. We selected the C-terminal antibody to avoid detecting the C-terminal truncated form of FcεRI-β(β_T_), which is known to have an alternative effect for FcεRI functions (Donnadieu et al., 2003). A single immunopositive band around 27 kDa with human basophils or mast-cell lysate was observed by Western blotting with the anti-FcεRI-β antibody and it did not react with eosinophil lysate (Fig. 1A). That result is reasonable because FcεRI-β expression is abundant in mast cells and basophils, but not in eosinophils (Seminario et al., 1999). A neutralization experiment with the FcεRI-β antibody preabsorbed with the immunized peptide completely abolished the 27 kDa band (Fig. 1B). We also confirmed the specificity by Western blotting using the preimmune serum, which did not show any bands with mast-cell lysate (data not shown). Next, we carried out co-immunoprecipitation of the FcεRI-αβγ complex using the anti-FcεRI-α monoclonal antibody with a lysis buffer containing phospholipids as previously reported (Rivnay et al., 1982) (Fig. 1C, D). The specificity of the FcεRI-β antibody was further verified because it did react with the co-immunoprecipitated FcεRI-αβγ complex by immunoblotting.

Immunocytochemical staining and FACS analysis showed that the FcεRI-β protein was expressed in the cell membrane and cytoplasm of LAD2 cells (Fig. 2A and B). The FcεRI-α chain protein was expressed on the cell surface and did not need cell permeabilization for staining (Fig. 2B, upper right), but the anti-FcεRI-β antibody did need cell permeabilization for staining (Fig. 2B, upper left). Therefore, we immunostained LAD2 cells in successive manners (the first incubation with the anti-FcεRI-α antibody was the non-permeabilized manner, while the second incubation with the anti-FcεRI-β antibody was carried out after saponin treatment). The double-immunostaining (Fig. 2B, lower right) showed colocalization of FcεRI-α and FcεRI-β (yellow color) underneath the cell cell-surface FcεRI-α staining (red color). These results seemed to be reasonable because this anti-FcεRI-β chain antibody was raised against the cytoplasmic portion of FcεRI-β protein. Although the exact subcellular localization of cytoplasmic FcεRI-β is still unknown, a previous report showed that FcεRI-αβγ receptor complex were was processed at endoplasmic reticulum (ER) and Golgi (Albrecht et al., 2000), so the cytoplasmic part of our FcεRI-β staining in LAD2 cells may reside in ER and Golgi. We also confirmed the FcεRI-α and FcεRI-β staining pattern in bone bone-marrow progenitor-cell-derived mast cells (b-mast cells: Fig. 3A), and specificity of immunocytostaining was further verified both with permeabilized and non-permeabilized eosinophils (negative controls: Fig. 3B).

As a next step, we showed that this FcεRI-β antibody is useful for quantification of the FcεRI-β protein within the 2.1 × 10^4^ to 1.5 × 10^5^ LAD2 cell range (Fig. 4A), making it possible to evaluate quantitatively the effect of monomeric IgE sensitization for the β-chain protein expression. Coordinated upregulation of the FcεRI-β chain with the mature α-chain (a hallmark for cell-surface FcεRI expression) had been noted 24-hours after IgE stimulation (Fig. 4B). In addition, we also quantified FcεRI-α and FcεRI-β mRNA expression in response to monomeric IgE incubation and found that no apparent FcεRI-α or FcεRI-β mRNA was induced with IgE incubation. These results are consistent with the previous reports which showed FcεRI upregulation after monomeric IgE binding (Asai et al., 2001 and Yamaguchi et al., 1999), and that monomeric IgE incubation did not upregulate FcεRI-α and FcεRI-β promoter activity (Asai et al., 2001). Considering two previous reports which showed that FcεRI-β protein has a role as a stabilizer of FcεRI expression (Donnadieu et al., 2000), and that the FcεRI-βγ complex supports FcεRI-α insertion to ER and promotes cell-surface expression (Fiebiger et al., 2005), we supposed that an increased FcεRI-β protein expression in response to monomeric IgE contributes to allergic reaction through the FcεRI stabilizing role.

The roles of FcεRI-β protein in mast cells are still controversial. One previous report suggested its role as an amplifier (Lin et al., 1996), and another showed the role as an inhibitory molecule (Gimborn et al., 2005) for degranulation and cytokine expression. Further studies are necessary for elucidating the roles of FcεRI-β protein, and of genetic variants of the FcεRI-β gene over its protein-expression control. This antibody will be a useful tool for elucidating the pathophysiological significance of FcεRI-β chain expression in atopic diseases.
